# A Chalcone from Ashitaba (*Angelica keiskei*) Stimulates Myoblast Differentiation and Inhibits Dexamethasone-Induced Muscle Atrophy

**DOI:** 10.3390/nu11102419

**Published:** 2019-10-10

**Authors:** Minson Kweon, Hyejin Lee, Cheol Park, Yung Hyun Choi, Jae-Ha Ryu

**Affiliations:** 1 Research Institute of Pharmaceutical Sciences and College of Pharmacy, Sookmyung Women’s University, 100 Chungparo 47-Gil, Yongsan-Gu, Seoul 04310, Korea; minson-_-@nate.com (M.K.); u9698115@naver.com (H.L.); 2 Department of Molecular Biology, College of Natural Sciences, Dong-eui University, Busan 47340, Korea; parkch@deu.ac.kr; 3 Department of Biochemistry, Dong-eui University College of Korean Medicine, Busan 47227, Korea

**Keywords:** Ashitaba, *Angelica keiskei*, 4-hydroxyderricin, myogenesis, muscle atrophy

## Abstract

Ashitaba, *Angelica keiskei* Koidzumi (AK), as a traditional medicine in Korea, Japan, and China, has been known as an elixir of life having therapeutic potential. However, there is no scientific evidence to support that Ashitaba can enhance or maintain muscle strength. To find a new therapeutic agent from the medicinal plant, we evaluated the anti-myopathy effect of chalcones from ethanol extract of AK (EAK) in cellular and animal models of muscle atrophy. To examine anti-myopathy activity, EAK was treated into dexamethasone injected rats and muscle thickness and histopathological images were analyzed. Oral administration of EAK (250 or 500 mg/kg) alleviated muscle atrophic damages and down-regulated the mRNA levels of muscle-specific ubiquitin-E3 ligases. Among ten compounds isolated from EAK, 4-hydroxyderricin was the most effective principle in stimulating myogenesis of C2C12 myoblasts *via* activation of p38 mitogen-activated protein kinase (MAPK). In three cellular muscle atrophy models with C2C12 myoblasts damaged by dexamethasone or cancer cell-conditioned medium, 4-hydroxyderricin protected the myosin heavy chain (MHC) degradation through suppressing expressions of MAFbx, MuRF-1 and myostatin. These results suggest that the ethanol extract and its active principle, 4-hydroxyderricin from AK, can overcome the muscle atrophy through double mechanisms of decreasing muscle protein degradation and activating myoblast differentiation.

## 1. Introduction

With the increasing population of older individuals, incidence rates of various chronic diseases are also increasing. Chronic conditions and aging can lead to skeletal muscle wasting and weakness, i.e., muscle atrophy. Cachexia is a muscle wasting condition generated by chronic diseases, such as cancer, kidney failure, and diabetes. More than 30% of pancreatic cancer patients have died of muscle atrophy accompanied by resistance to chemotherapy and therapy-related side effects [1,2]. In addition, 50% of individuals over 80 years of age who suffer from fragility fractures have sarcopenia, an age-related muscle wasting condition [3]. Although the severity of muscle atrophy is increasing, only megestrol acetate has been approved by U.S. FDA for the treatment of cancer and AIDS induced cachexia. However, megestrol acetate has shown side effects such as insomnia, skin rash, impotence and fever. Thus, new agents having anti-myopathy potential with less side effects are needed to overcome the disease and age-associated muscle atrophy.

Muscle atrophy can be caused by impaired muscle regeneration following decreased number and dysfunction of muscle satellite cells (referred to as muscle stem cell) and elevated degradation of muscle protein [1]. Dexamethasone (a synthetic glucocorticoid) or conditioned media (CM) from cancer cell culture can lead muscle atrophy. They increase NF-κB-mediated expression of muscle specific E3 ligases such as muscle atrophy F-box (MAFbx) and muscle-specific RING finger protein-1 (MuRF1), which are associated with ubiquitin-proteasomal degradation of myofibers [4,5,6]. The dexamethasone-induced muscle atrophy in Sprague–Dawley rats was a well-established animal model to find therapeutic agents for muscle atrophy under several wasting conditions [7].

*Angelica keiskei* Koidzumi (AK) (Japanese name “Ashitaba” meaning tomorrow leaf, Korean name “Shinsuncho” meaning elixir of life, Umbelliferae) has been used as a traditional medicine [8] and diverse dietary supplements of Ashitaba tea or juice were prepared. The inhabitants of Hachijo-jima, a village famous for longevity in Japan, believe that Ashitaba has been improving their health [9]. Roots and leaves of Ashitaba were known to be effective for improving asthma, chronic hepatitis, diabetes, gastritis, high blood pressure, obesity, and psoriasis. In addition, Ashitaba has been transmitted in folk remedy in Asian countries as a treatment for muscle and joint pain.

As chalcones of Ashitaba, including 4-hydroxyderricin (4-HD), isobavachalcone, xanthokeismin A, and xanthoangelol B, E, D, and F have been reported to possess a wide range of pharmacological activities including anti-oxidative and anti-inflammatory potential. Especially, two chalcones, 4-HD and xanthoangelol have attracted attention to develop herbal supplements or medicines due to their pharmacological potentials and high contents in this edible herb [10,11]. Recently, metabolomic and lipidemic analyses of human plasma revealed that five components of AK including 4-HD are responsible for preventive effects against liver diseases, type 2 diabetes, obesity and atherosclerosis [12]. Studies on toxicity and metabolism of 4-HD and xanthoangelol were published in rat or human models. These reports encourage their pharmaceutical applications [13,14]. The regulatory effect of two chalcones on glucose metabolism in muscle [15] implies their potential application for muscle strength, regarding the beneficial relationship between muscle hypertrophy and glucose metabolism [16].

In this study, we identified a chalcone compound, 4-HD as the most potent myogenesis-stimulating agent among compounds purified from AK. We investigated the preventive and protective effects of AK extract and 4-HD against muscle atrophy both in vivo and in vitro models and disclosed underlying action mechanisms.

## 2. Materials and Methods

### 2.1. Animals

Sprague–Dawley rats (6 weeks old, male) were purchased from Samtako (Osan, Korea). They were maintained in controlled environment (23 ± 1 °C, 55 ± 5% relative humidity) under a 12 h light/dark cycle for acclimation. Rats were group housed in poly-propylene cages (3 rats per cage) and were provided *ad libitum* access to water and a standard laboratory diet. All animal experiments were conducted according to the National Institutes of Health Guide for the Care and Use of Laboratory Animals (8th edition, revised in 2011) and approved by the Institutional Animal Committee of Dong-eui University (#A2017-006/2017).

### 2.2. Experimental Procedures

After 8 days acclimation, these rats (mean weight was 253 g) were randomly divided into five groups: (i) the intact vehicle control group, (ii) dexamethasone (Dex) control group, (iii) Dex and ethanol extract of *A. keiskei* (EAK) 250 mg/kg-treated group, (iv) Dex and EAK 500 mg/kg-treated group, and (v) Dex and oxymetholone-treated group (6 animals per group). Different groups were randomly treated in each experiment. To induce muscle atrophy, Dex (1 mg/kg body mass) was intraperitoneally injected daily for 7 days. Two different concentrations of EAK (250 and 500 mg/kg body mass) were orally administered once daily for 28 days. Oxymetholone (50 mg/kg body mass) was administered orally for the same period as EAK administration. Oxymetholone is a synthetic anabolic steroid used as an agent to accelerate muscle growth and shows ameliorative effects on muscle wasting after dexamethasone treatment in animal models [17].

### 2.3. Measurement of Body Weight and Gastrocnemius Muscle Thickness

Body weight was measured at the end of test material administration using an automatic electronic balance machine (Precisa Instruments, Dietikon, Switzerland). A 25 mg/kg of zoletil (Zoletil 50^®^; Virbac, Nice, France) was injected into the abdominal cavity of each rat at the end of test material administration and the gastrocnemius muscle (GA) was exposed. The thickness of exposed GA of the left hind limb was measured using an electronic digital caliper (Mytutoyo, Tokyo, Japan) and photographed.

### 2.4. Histopathological Analysis

Samples of gastrocnemius muscle were separated, fixed in 10% neutral-buffered formalin for 24 h, embedded in paraffin, transversely sectioned (3 μm in thickness), and then stained with hematoxylin and eosin (H&E) for general histopathological analysis. Histopathological profiles of each sample were then determined by light microscopy observation (Nikon, Tokyo, Japan).

### 2.5. Preparation of Chalcones from Roots of AK

Dried roots of AK were purchased from Sengdong-nongsan Co. (Chungju, Korea) and authenticated by Prof. Kisook Yang at the College of Pharmacy, Sookmyung Women’s University. A voucher specimen (No. SPH 1501) was deposited at the Herbarium of Sookmyung Women’s University. Chalcones were isolated from ethanol extract (26 g) of EAK as previously reported and dissolved in dimethyl sulfoxide for treating cells [10]. All chemical structures were elucidated by spectroscopic data analysis followed by comparison with previously described data [10,18], and the purities (>96%) were determined by ^1^H-NMR spectra and HPLC. The HPLC analysis was performed using Waters 1525 system (Milford, MA, USA). A reverse phase column (ODS-2, 5 μm, 150 × 4.6 mm i.d., GL Sciences Inc., Tokyo, Japan) was eluted with 80% methanol (flow rate 1 mL/min), and monitored with PDA detector at 360 nm. Copies of the original spectra are obtainable from the corresponding author.

### 2.6. Cell Culture, Myoblast Differentiation and Preparation of Conditioned Medium of CT26 Cancer Cells

C2C12 myoblast cells obtained from the American Type Culture Collection (ATCC, Manassas, VA, USA) were differentiated for 3 days as demonstrated in our previous studies [19]. We prepared CT26 murine colon carcinoma cell-conditioned medium (CM) according to previous studies [19]. To prepare 4-HD-treated CM (HD-CM), CT26 cells were plated and treated with 4-HD for 24 h in DMEM containing 10% FBS. After washing with PBS, cells were incubated in serum free DMEM for another 24 h. The prepared CM was diluted with fresh differentiation medium (DMEM containing 2% horse serum) to make a final concentration of 30% for treating myoblasts or myotubes.

### 2.7. MyoD-Reporter Gene Assay

To measure MyoD transcriptional activity, C2C12 cells were transiently transfected with MyoD-responsive reporter 4RTK-luciferase (RTK-Luc) and pBP-MyoD constructs using Lipofectamine^®^ 2000 Reagent (Invitrogen, Carlsbad, CA, USA) [19]. At 24 h post transfection, cells were treated with test compounds for 24 h. Luciferase assay was then performed using cell lysates and a commercial luciferase assay kit (Promega, Madison, WI, USA). Data are reported as relative luciferase activity (RLU) divided by β-galactosidase activity.

### 2.8. Immunostaining of Myosin Heavy Chain (MHC)

In brief, treated myoblasts or myotubes were fixed, permeabilized, and incubated with a primary antibody against MHC (MAB4470, R&D Systems, Minneapolis, MN, USA) at 4 °C overnight followed by incubation with a goat anti-mouse antibody conjugated with Alexa Fluor 568 (Life Technologies, Carlsbad, CA, USA) [20]. These cells were counterstained with DAPI (4′,6-diamidino-2-phenylindole, Sigma-Aldrich). MHC immunofluorescence was then detected under a fluorescence microscope (Olympus, Tokyo, Japan). A red fluorescence indicates MHC expression while multinucleated myotubes are observed with DAPI (blue-colored) counterstaining. The number of MHC-expressing multinucleated (containing more than four nuclei) myotubes in the counting field was presented as a relative change to that of control group. Images were captured with a Nikon ECLIPSE TE-2000U microscope and processed with NIS-Elements F software (Nikon) and Photoshop CS5 (Adobe).

### 2.9. Western Blot Analysis

C2C12 cells were differentiated in DM with the test sample and cell lysates were subjected to Western blotting analysis to determine protein expression of myogenic markers and E3 ligases. Harvested cells were suspended in lysis buffer (50 mM Tris, pH 7.4, 150 mM NaCl, 10% glycerol, 1.5 mM MgCl_2_, 1 mM EGTA, 1% Triton X-100, 10 mM NaF, 1 mM Na_3_VO_4_, and protease inhibitor cocktail) and proteins were loaded onto sodium dodecyl sulfate-polyacrylamide gel, electrophoresed, and transferred to polyvinylfluoride membrane in transfer buffer (39 mM glycine, 48 mM Tris base, 0.037% SDS, and 20% methanol) for 2 h. The membrane was then incubated with antibodies specific to MHC (Santa Cruz Biotechnology, Dallas, TX, USA), MyoD (Santa Cruz Biotechnology), Myogenin (Santa Cruz Biotechnology), MAFbx (Santa Cruz Biotechnology), and MuRF1 (Santa Cruz Biotechnology). p38 MAPK activation was analyzed by using antibodies against phospho-p38 (Cell Signaling Technology, Danvers, MA, USA) and p38 MAPK (Cell Signaling Technology). Pan-cadherin (Sigma, St. Louis, MO, USA) was used as a loading control. 

### 2.10. RNA Extraction and Reverse Transcription Polymerase Chain Reaction (RT-PCR)

Gene expression of E3 ligases was analyzed by RT-PCR. RNAs were extracted from myoblasts or myotubes using Trizol Reagent (Life Technologies). First-strand cDNA synthesis was then performed using Labopass™ cDNA synthesis kit (Cosmogenetech, Seoul, Korea) according to the manufacturer’s recommendation. PCR was carried out as follows: 5 min at 94 °C, 30 cycles of 94 °C for 1 min, 56 °C for 1 min, and 72 °C for 1 min, and a 5-min incubation at 72 °C. All mRNA levels were normalized to glyceraldehyde 3-phosphate dehydrogenase mRNA levels. The final PCR amplicons were then separated using electrophoresis with a 1.8% agarose gel. The primers used for the amplifications are shown in Table 1.

### 2.11. Statistical Analysis

All values are presented as mean ± standard deviation (SD). Differences were assessed using Student’s *t*-test or one-way analysis of variance (ANOVA) followed by Duncan’s test. All experiments were performed at least three times. Difference with a *p* value of less than 0.05 was considered statistically significant.

## 3. Results

### 3.1. Ethanol Extract of Angelica keiskei Alleviates Dexamethasone-Induced Muscle Atrophy in Rats

**Figure 1:**
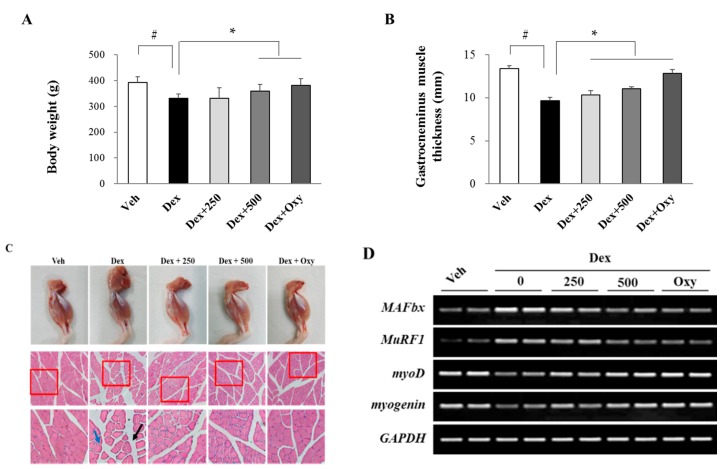
Anti-myopathy activity of ethanol extract of *Angelica keiskei* in dexamethasone-induced muscle atrophied rats. Effects of ethanol extract of AK (EAK) (250 and 500 mg/kg) on body weight (**A**) and gastrocnemius muscle thickness (**B**). (**C**) Gross appearance and histopathological images of the hind limb of rats. Black arrow indicates the perimysium and blue arrow indicates the endomysium. (**D**) Changes in gastrocnemius muscle mRNA expression were measured by reverse transcription polymerase chain reaction (RT-PCR). Oxymetholone (Oxy) was used as a positive control (50 mg/kg). Data are expressed as mean + SD (n = 6). # *p* < 0.005 vs. vehicle control rat (Veh); * *p* < 0.005 vs. dexamethasone treated rat (Dex).

To investigate the protective effect of ethanol extract of *Angelica keiskei* Koidzumi (EAK) against muscle atrophy induced by dexamethasone in rats, we measured body weight and gastrocnemius muscle thickness. As shown in Figure 1A,B, dexamethasone treatment decreased body weight and gastrocnemius muscle thickness compared with vehicle control. However, EAK treatment (500 mg/kg) significantly recovered the damage. As shown in histopathological staining of muscle (Figure 1C), dexamethasone increased thickness of perimysium and endomysium, and decreased muscle fiber diameters compared with the intact vehicle control. EAK ameliorated these atrophic damages in a dose-dependent manner. Next, we investigated the mechanism involved in EAK-mediated muscle protection in animal by measuring mRNA levels of muscle-specific E3 ligases. Dexamethasone significantly increased expressions of muscle RING finger protein-1 (MuRF1) and muscle atrophy F box (MAFbx/atrogin-1) in gastrocnemius muscles (Figure 1D). However, EAK (500 mg/kg) decreased these expression levels of MuRF1 or MAFbx mRNA, while it increased expression levels of MyoD and myogenin (myogenic factors). Oxymetholone, a representative 17α-alkylated anabolic-androgenic steroid, was known to inhibit the glucocorticoid-induced loss of body weight and muscle *via* its anabolic effects [17]. We used oxymetholone as a positive control in animal study (50 mg/kg).

### 3.2. EAK and Its Components Promote Myogenesis

**Figure 2:**
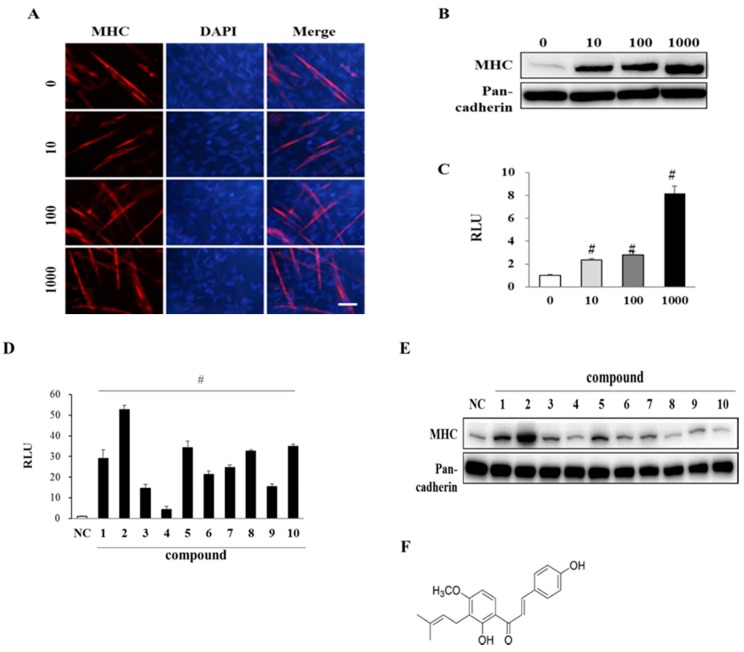
Effects of EAK and AK compounds on myogenesis of myoblasts. (**A**) C2C12 cells were differentiated in differentiation medium (DM) supplemented with EAK (10, 100, or 1000 ng/mL) for 3 days and immunostained with antibody to myosin heavy chain (MHC, red) and 4′,6-diamidino-2-phenylindole (DAPI, blue). Scale bar = 200 μm. (**B**) Expression level of MHC was determined by Western blotting analysis. (**C**) C2C12 cells were transiently transfected with 4RTK-luciferase and MyoD-pBP plasmid, and then treated with EAK as described in materials and methods. MyoD transactivation is presented as relative luciferase activity (RLU). Data are expressed as mean ± standard deviation (SD). # *p* < 0.001 vs. 0 ng/mL of EAK. (**D**) C2C12 cells were transiently transfected with 4RTK-luciferase and MyoD-pBP plasmid, and then treated with AK compounds **1**–**10** (100 pM) for an additional 24 h. Data are expressed as mean ± standard deviation (SD). NC, negative control (DMSO); **1**–**10**, compounds 1–10. # *p* < 0.001 vs. NC. (**E**) C2C12 cells were differentiated in the presence of each AK compounds **1**–**10** and then expression level of MHC was determined by Western blotting analysis. (**F**) Chemical structure of 4-HD.

Mouse skeletal myoblast C2C12 is derived from mouse satellite cell and well-established cell line to study the myogenic potential of chemicals [21]. C2C12 myoblasts were differentiated with EAK (10, 100, or 1000 ng/mL), which dose-dependently enhanced expression levels of MHC (Figure 2A,B) as a marker of terminal myogenesis [19]. We observed the cylinder-shaped multinucleated myotubes (containing more than four nuclei and indicating mature myotubes) by immunostaining for MHC and DAPI [19]. The number of multinucleated myotubes was increased by EAK in a dose-dependent manner. We also evaluated myogenic activities of EAK by measuring MyoD transcriptional activities using a reporter gene assay. EAK dose-dependently increased MyoD transcriptional activities (Figure 2C).

EAK was partitioned with ethyl acetate and water to isolate myogenic principles. Ethyl acetate-soluble fractions were subjected to silica gel column chromatography from which ten chalcones were purified: isobavachalcone (**1**), 4-hydroxyderricin (4-HD) (**2**), xanthoangelol E (**3**), xanthoangelol D (**4**), xanthoangelol (**5**), xanthoangelol F (**6**), xanthokeismin A (**7**), 1-[2,4-dihydroxy-3-(6,7-dihydroxy-3, 7-dimethyl-2-oxtenyl)phenyl] 3-(4-hydroxyphenyd, l-2-prepen-1-one chalcone (**8**), 1-[2-hydroxy-3-(7-hydroxy-3,7-dimethyl-2,5-ocadienyl)-4-methoxyphenyl]-3-(4-hydroxyphenyl)-2-propen-1-one-chalcone (**9**), and xanthoangelol B (**10**). Their chemical structures were identified by spectroscopic analysis and comparison with previous data [18,22].

All AK compounds increased MyoD transcriptional activity (Figure 2D) and myosin heavy chain (MHC) expressions in 100 pM concentrations (Figure 2E). The effective dose of compounds was unexpectedly very low. With considering the effective dose in our animal study (500 mg/kg), the in vitro concentration could not be extrapolated to effective dose in in vivo study. Since 4-HD (compound **2**, Figure 2F) showed the strongest activity among them in MyoD transactivation and MHC expression, we used this compound in further study. The main constituents of EAK were identified as 4-HD and xanthoangelol (compound **5**) by HPLC, and their contents were quantified as 2.8% and 4.8% (w/w) respectively (data not shown).

### 3.3. 4-HD Stimulates Myogenesis

**Figure 3:**
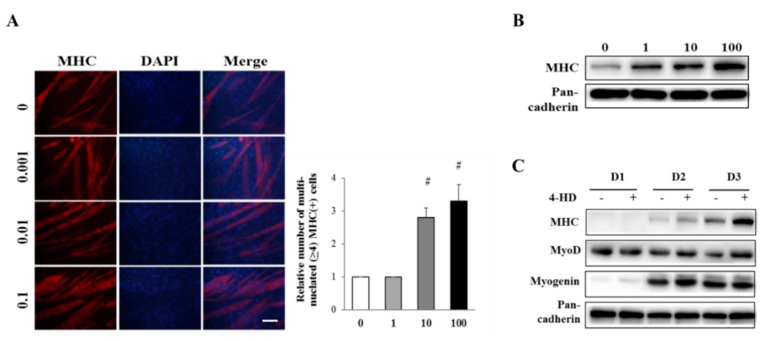
Effect of 4-HD on myogenesis of myoblasts. (**A**) Differentiated C2C12 cells in the presence of 4-HD (1, 10, 100 pM) were immunostained with antibody to MHC (red) and 4′,6-diamidino-2-phenylindole (DAPI, blue). Multinucleated (>4 nuclei) and MHC-expressing myotubes were counted from randomly selected areas. Scale bar = 200 μm. # *p* < 0.005 vs. 0 nM. (**B**) Expression levels of MHC were determined by Western blotting analysis. (**C**) C2C12 myoblasts were treated with 4-HD (100 pM) during myogenesis period (D1, D2, D3) and levels of myogenic factors were measured by Western blotting analysis.

To examine the myogenic effect of 4-HD, C2C12 myoblasts were differentiated in the presence of 4-HD. As shown in Figure 3, 4-HD dose-dependently enhanced expression of MHC and the number of multinucleated myotubes in sub-nanomolar concentrations. These data demonstrate that picomolar concentration of 4-HD is sufficient to induce myoblast differentiation from C2C12 myoblasts.

Expression levels of MHC and myogenin were gradually increased during differentiation period (Figure 3C), and were further increased by 4-HD treatment (100 pM). The expression level of MyoD, as an initiator of myogenesis, was gradually decreased after DM treatment. However, 4-HD treatment maintained MyoD level during myogenesis compared with the respective control myoblasts.

### 3.4. 4-HD Stimulates Myogenesis by p38 MAPK Activation

**Figure 4:**
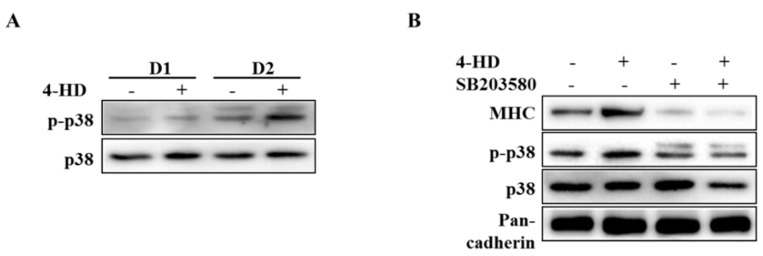
Effect of 4-HD on p38 MAPK activation during myogenesis. (**A**) 4-HD (100 pM) treated myoblasts were harvested at indicated differentiation periods. (**B**) Confluent myoblasts were pre-incubated with SB203580 (10 μm) for 1 h, followed by 4-HD stimulation for 2 days. The phospho-p38 MAPK was measured by Western blotting analysis.

To determine the mechanism involved in the myogenic activity of 4-HD, we examined activation of p38 mitogen-activated protein kinase (MAPK) that was reported to activate myoblast differentiation [23]. As shown in Figure 4A, basal level of phospho-p38 MAPK was gradually increased to drive myogenesis. 4-HD further increased the level of phospho-p38 MAPK during myogenesis. To clarify the role of p38 MAPK in 4-HD-induced myogenesis, C2C12 myoblasts were pretreated with 10 μm of SB203580, a pharmacological inhibitor of p38 MAPK, ahead of 4-HD. SB203580 reduced basal expression of MHC and also suppressed 4-HD induced-MHC expression to basal level (Figure 4B).

### 3.5. 4-HD Protects Against Muscle Wasting In vitro

**Figure 5:**
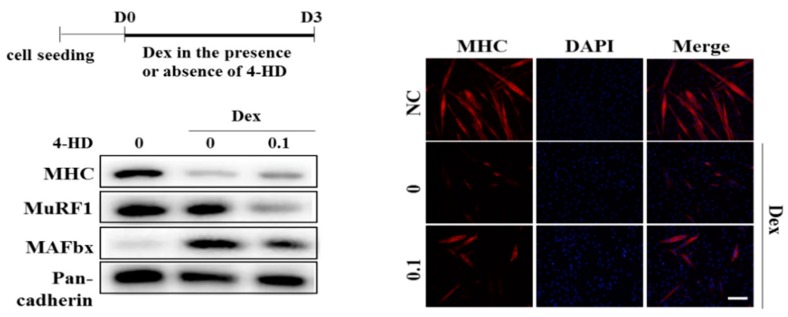
Protective effect of 4-HD against muscle wasting in vitro. C2C12 Cells were treated according to the scheme and expression levels of MHC and muscle E3 ligases were determined by Western blotting analysis. MHC and DAPI were immunostained in myotubes. Scale bar = 200 μm.

To evaluate a protective effect of 4-HD, we adopted three types of muscle wasting models by using dexamethasone or conditioned media (CM) of colon cancer cells. Firstly, C2C12 myoblasts were differentiated with DM containing dexamethasone (20 μM) in the presence or absence of 4-HD for 3 days. Dexamethasone, a synthetic glucocorticoid, was reported to induce muscle atrophy in vitro and in vivo [17,24]. Dexamethasone significantly decreased MHC expression and multinucleated myotubes, and enhanced MAFbx expression, while 4-HD (100 pM) decreased the dexamethasone-induced MHC loss and MAFbx expression. The level of MuRF1 was significantly reduced by 4-HD treatment compared with dexamethasone group, even though it was not significantly enhanced by dexamethasone treatment (Figure 5).

**Figure 6:**
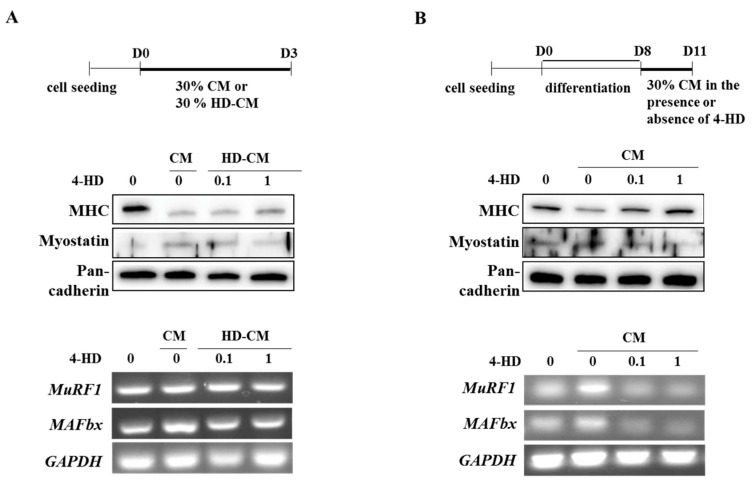
Preventive and protective effects of 4-HD against muscle wasting in vitro. Myoblasts (**A**) and myotubes (**B**) were treated according to the scheme shown, and expression levels of MHC, myostatin, and muscle E3 ligases were determined by Western blotting analysis or RT-PCR.

Secondly, differentiation of C2C12 myoblasts were induced into myotubes for 3 days with 30% conditioned media (CM) or 4-HD (0.1 or 1 nM) treated CM (HD-CM) (Figure 6A). As expected, CM decreased MHC and increased myostatin protein levels compared with DM control. However, 30% HD-CM reversed levels of MHC and myostatin that might come from the decreased release of atrophic factors from cancer cells by 4-HD treatment. CM increased mRNA levels of *MAFbx* compared with DM control whereas HD-CM blunted *MAFbx* expression. However, mRNA expression of *MuRF1,* another E3 ligase, was not affected by 4-HD treatment.

Thirdly, myoblasts were differentiated for 8 days followed by incubation with 30% CM to induce myotube damage. CM significantly decreased MHC, increased myostatin protein, and increased mRNA expression of E3 ligases in myotubes. However, 4-HD reversed such effects of CM in a dose-dependent manner. 4-HD protected against CM-induced MHC degradation by suppressing expression of E3 ligases (*MAFbx* and *MuRF1*) and myostatin compared with CM control (Figure 6B). These results suggest that 4-HD may protect muscle wasting in cancer-induced cachexia condition.

## 4. Discussion

Ashitaba, *Angelica keiskei* Koidzumi (AK), has been used as both a vegetable and folk medicine in Asian countries. Recently many dietary supplements were developed using Ashitaba for heartburn, stomach ulcers, high blood pressure, high cholesterol, gout, and constipation. Ethanol extracts of AK and the main ingredients (4-hydroxyderricin and xanthoangelol) were reported to regulate glucose uptake and improve insulin sensitivity in muscle in vivo or in vitro models [25]. Considering the positive effects of AK extract and main ingredients on glucose metabolism in skeletal muscle, we hypothesized that AK could improve physical activities as well as muscle strength. In our preliminary study, EAK (250, 500 mg/kg) significantly extended running time of mice in treadmill test as compared with dexamethasone-damaged groups (Figure S1). So, we tried to find out the active constituent of EAK and disclose the underlying mechanism of action.

Muscle quiescent satellite cells can be activated by exercise or muscle injury to regenerate and repair injured muscles. Activated myogenic satellite cells undergo myogenesis to become myoblasts (proliferating satellite cells), and then differentiated into mature myotubes. During myoblast differentiation, various myogenic factors such as MyoD, myogenic factor (Myf)-5, myogenin, myogenic regulatory factor (MRF)-4, and myosin heavy chain (MHC), not only contribute to myogenic lineage specification of muscle stem cells, but also initiate and terminate myoblast differentiation [26,27].

Considering the role of satellite cells, we purified ten chalcones from AK, which has been used as food and herbal medicine, and we identified activators of satellite cells as potential therapeutic agents for muscle atrophy. Among tested chalcones, 4-hydroxyderricin (4-HD) was the most potent myogenic constituent of AK. At the same concentration (10 ng/mL), 4-HD showed a 15-fold transcriptional activation of MyoD compared with EAK. In the literature, 4-HD has been reported to exhibit anti-inflammatory, anti-microbial, anti-platelet, anti-diabetic, and anti-obesity activities [11,28]. The present study demonstrated that 4-HD stimulates myogenesis in dose- and time-dependent manners *via* p38 mitogen-activated protein kinase (MAPK) activation. The p38 MAPK phosphorylates E proteins, SWI/SNF subunit BAF60, or Mef2 to form complex with MyoD which in turn initiate the expression of myogenic factors [29]. As p38 MPAK activation is an essential mechanism for myogenesis [23], we observed activation of p38 MAPK by 4-HD during myogenesis, while SB203580 (a p38 MAPK inhibitor) abrogated the myogenic effects of 4-HD, implying the central role of p38 MAPK in 4-HD-induced myogenesis. In this study, we report for the first time the myogenic potential of 4-HD and AK that can be applied to treatment of muscle atrophy.

Another considerable therapeutic strategy for muscle atrophy is inhibition of muscle protein degradation. Under cachexic conditions, myo-proteins undergo ubiquitin modification by E3 ubiquitin ligases and proteasome-dependent degradation [5,6]. Thus, increased expression of E3 ubiquitin ligases indicate the progress of muscle atrophy accompanied by myofibrillar and intracellular proteins breakdown [17,30]. Bodine et al. have shown that MAFbx or MuRF1-deficient mouse can overcome muscle atrophy, whereas MAFbx overexpressing myoblasts exhibit retardation in myotube formation [31]. Although inhibitors of proteasomal degradation such as bortezomib and MG132 can attenuate myo-protein degradation, their application for treating muscle diseases has not been reported yet.

Myostatin, a negative regulator of skeletal muscle growth, contributes to the loss of muscle mass associated with aging and muscle diseases. In a recent study, knockouts (Mst-/-) or spontaneous mutation of myostatin induced a hypermuscular phenotype in mice and humans [32,33]. Therefore, myostatin inactivation may prevent muscle loss in muscular atrophy.

In order to find the muscle protecting potential of 4-HD, we used three kinds of in vitro models to mimic muscle atrophy. In these models, we observed retardation of myogenesis and loss of myotubes together with the reduction of MHC and the increment of myostatin and E3 ligase expressions. It was shown that 4-HD reversed these atrophic conditions by showing increased MHC and decreased myostatin and E3 ligase. The anti-inflammatory potential of 4-HD might have alleviated CM-induced inflammatory environment to stimulate myogenesis [28].

Previous studies have shown that glucocorticoids can induce muscle atrophy through physiological muscle changes in rodents [5,6]. Glucocorticoids can induce catabolic muscle atrophy accompanied by characteristic histopathological changes, including loss of cell organelles, decrease of protein content and muscle fiber diameter [34,35]. In the present study, we evaluated protective effects of ethanol extract of AK (EAK) against dexamethasone-induced muscle degradation in animal experiment. Dexamethasone reduced gastrocnemius muscle thickness, muscle fiber diameters, and body weight of rats, while EAK significantly improved these characteristics of muscle atrophy. EAK also blunted the expression of muscle specific E3 ligases (MuRF1 and MAFbx) in the gastrocnemius muscle of dexamethasone treated rats.

## 5. Conclusions

In summary, the ethanol extract and its active principle, 4-hydroxyderricin from Ashitaba, can overcome the muscle atrophy through double mechanisms of decreasing muscle protein degradation and activating myoblast differentiation. It was shown that 4-hydroxyderricin alleviates muscle atrophy by stimulating myogenesis *via* p38 MAPK activation and protecting muscle waste *via* down-regulation of E3 ligases (MuRF1 and MAFbx) and myostatin. In muscle atrophic rat model, ethanol extracts of *Angelica keiskei* Koidzumi protected the loss of body weight and gastrocnemius muscle mass through suppressing the expression of muscle-specific E3 ligases. Taken together, ethanol extract of *A. keiskei* and 4-hydroxyderricin have therapeutic potential for treating muscle atrophy.

## Figures and Tables

**Table 1:** Oligonucleotide primer sequences used for the RT-PCR analysis.

Gene Name	Forward Primer	Reverse Primer	Accession Number
*MAFbx* *MuRF1* *GAPDH*	CGACCTGCCTGTGTGCTTAC GGTGCCTACTTGCTCCTTGT TGCACCACCAACTGCTTAG	CTTGCGAATCTGCCTCTCTG CTGGTGGCTATTCTCCTTGG GGCATGGACTGTGGTCATGAG	BC027211 NC_000070 BC0960 42

*MAFbx*, muscle atrophy F-box (MAFbx/atrogin-1); *MuRF1*, muscle RING finger protein-1; *GAPDH*, glyceraldehyde 3-phosphate dehydrogenase.

## Supplementary Materials

The following are available online at https://www.mdpi.com/2072-6643/11/10/2419/s1, Figure S1: Effect of ethanol extract of *Angelica keiskei* (EAK) on exercise endurance in dexamethasone-induced muscle atrophied mice.
