# Supplementary material for: A Chalcone from Ashitaba (Angelica keiskei) Stimulates Myoblast Differentiation and Inhibits Dexamethasone-Induced Muscle Atrophy
Source: Nutrients. 2019 Oct 10;11(10):2419. doi: 10.3390/nu11102419 (PMC6835314; doi:10.3390/nu11102419)
Supplement: Supplementary file 1 [file nutrients-11-02419-s001.pdf]

A

	Veh	500	Dex	Dex + 250	Dex + 500	Dex + Oxy	*P*-value
**Distance (m)**	810.7 ± 34.3[a]	821.0 ± 32.6[a]	752.8 ± 64.7[b]	805.8 ± 65.6[a]	802.0 ± 65.8[a]	816.9 ± 30.5[a]	< 0.049

B

	Veh	500	Dex	Dex + 250	Dex + 500	Dex + Oxy	*P*-value
**Gastrocnemius (g)**	1.06 ± 0.10	1.14 ± 0.11	0.81 ± 0.08	0.86 ± 0.15	0.89 ± 0.15	0.89 ± 0.06	0.175
**Soleus (g)**	1.06 ± 0.03	0.19 ± 0.04	0.13 ± 0.03	0.15 ± 0.05	0.16 ± 0.03	0.15 ± 0.10	0.159
**Total muscle (g)**	1.22 ± 0.13	1.33 ± 0.15	0.94 ± 0.11	1.01 ± 0.20	1.05 ± 0.18	1.04 ± 0.07	0.052

C

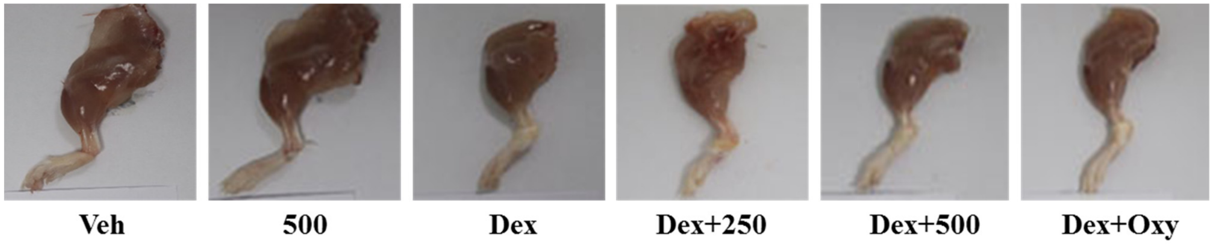

Figure S1. Effect of ethanol extract of *Angelica keiskei* (EAK) on exercise endurance in dexamethasone-induced muscle atrophied mice. Effects of EAK on (A) Running endurance time in treadmill test and (B) Muscle weight. (C) Representative photographs of isolated gastrocnemius muscle from mice. Different amounts of EAK (250 and 500 mg/kg) were orally administered for 28 days. Muscle atrophy induced by a subcutaneous injection of dexamethasone (1 mg/kg) for 10 days. Oxymetholone (Oxy) was used as a positive control (50 mg/kg). Data are

expressed as mean ± SD of 6 mice. Significance of differences between groups were determined using the one-way analysis of variance (ANOVA). Veh, vehicle; Dex, dexamethasone; Oxy, oxymetholone.
